# Prevalence and socio-economic burden of heart failure in an aging society of South Korea

**DOI:** 10.1186/s12872-016-0404-2

**Published:** 2016-11-10

**Authors:** Hankil Lee, Sung-Hee Oh, Hyeonseok Cho, Hyun-Jai Cho, Hye-Young Kang

**Affiliations:** 1 College of Pharmacy, Yonsei Institute of Pharmaceutical Sciences, Yonsei University, Incheon, South Korea; 2 Department of Internal Medicine, Division of Cardiology, Seoul National University Hospital, Seoul, South Korea

**Keywords:** Heart failure, Economic burden, Cost of heart failure, South Korea, Sensitivity analysis

## Abstract

**Background:**

Heart failure (HF) is one of the leading causes of morbidity and mortality in South Korea. With the rapidly aging population in the country, the prevalence of HF and its associated costs are expected to rise continuously. This study was carried out to estimate the prevalence and economic burden of HF in order to understand its impact on our society.

**Methods:**

A prevalence-based, cost-of-illness study was conducted using the 2014 Health Insurance Review and Assessment Service-National Patients Sample (HIRA-NPS) data. Adult HF patients were defined as those aged ≥19 years who had at least one insurance claim record with a primary or secondary diagnosis of HF (ICD-10 codes of I11.0, I13.0, I13.2, and I50.x). The costs consist of direct costs (i.e., medical and non-medical costs) and indirect costs (i.e., productivity loss cost due to morbidity and premature death). Subgroup analyses were conducted by age group, history of HF hospitalization, and type of universal health security program enrolled in.

**Results:**

A total of 475,019 adults were identified to have HF in 2014. The estimated prevalence rate of HF was 12.4 persons per 1,000 adults. According to the base cases and the extended definition of the cases, the annual economic burden of HF from a societal perspective ranges from USD 1,414.0 to 1,560.5 for individual patients, and from USD 752.8 million to 1,085.6 million for the country. A high percentage (68.5 %) of this socioeconomic burden consist of medical costs, followed by caregiver’s cost (13.2 %), productivity loss costs due to premature death (10.8 %) and morbidity (4.2 %), and transportation costs (3.4 %). The HF patients with prior hospitalization due to HF annually spent 9.7 times more for National-Health-Insurance-covered medical costs compared to HF patients who were not previously hospitalized.

**Conclusions:**

In the present study, HF patients who were older and had a history of prior hospitalization for HF as well as an indigent status were shown at high risk of spending more for healthcare to treat their HF. An effective disease management protocol should be employed to target this patient group.

## Background

According to the American College of Cardiology Foundation and American Heart Association [1], heart failure (HF) is defined as “a complex clinical syndrome that results from any structural or functional impairment of the ventricular filling or ejection of blood”. More than two-thirds of HF patients have underlying diseases such as ischemic heart disease, chronic obstructive pulmonary disease, hypertensive heart disease, and rheumatic heart disease [2]. The early symptoms of HF include chronic fatigue, indigestion, insomnia, and headache. Based on the progression of the disease, swelling, ascites, and dyspnea due to lung congestion are commonly developed.

HF is a progressive disease with repeated recurrences and improvements, resulting in frequent hospitalization [3]. It has been observed in Minnesota, U.S. that about 16.5 % of HF patients experience at least one hospitalization associated with HF during their lifetime, and that 83.1 % are hospitalized for all types of reasons [4]. Roughly one in four HF patients among the Medicaid beneficiaries in the U.S. is readmitted within a month after discharge with HF [5]. In particular, among those 65 years or older, HF is the most common reason for hospitalization [6]. Whellan et al. reported that 66 % of the elderly in their study who were hospitalized due to HF were readmitted for HF in the following year [7]. The numerous symptoms of and repeated hospitalizations for HF negatively affect the patient’s quality of life and increase the patient’s economic burden [8]. In Sweden, the annual economic burden attributed to HF was SEK 2.0–2.6 billion, accounting for 2 % of Sweden’s public healthcare budget [9]. In England, the direct medical costs for HF treatment accounted for 1.9 % of the entire National Health Service budget [10]. Since the onset of HF is strongly correlated with aging [2], the prevalence of HF is expected to grow worldwide with the aging population trend.

In South Korea, the number of patients with HF has been increasing in recent years, with an average annual increase rate of 4.5 % from 2009 to 2013. In those aged 80 years or above, the reported annual increase rate is 9 %, which is about twice that of the adult population [11]. In addition, HF is one of the leading causes of death in South Korea [12]. The aging population rate in Korea is on a very steep rise, and the percentage of the population aged 60 years and above is predicted to increase from 13.7 % in 2015 to 28.6 % by 2050 [13]. With such a rapidly aging population in South Korea, it is expected that the prevalence of HF and its associated costs will continue to grow. One useful approach to supporting the rationale of allocating healthcare resources to a specific condition would be to provide information on the extent to which the patients themselves and our society suffer from the burden of that condition. Thus, in the present study, the prevalence of HF was estimated, and the impact of HF on the South Korean society was determined by estimating the economic burden of HF from the perspectives of the National Health Insurance (NHI) and the society. Understanding the burden of HF and identifying the patient subgroups with a higher economic burden will aid in the prioritization of healthcare resource allocation.

## Methods

### Study design and data source

The economic burden of HF was estimated based on a “prevalence-based approach,” which measured the costs associated with treating HF among both new and pre-existing cases of HF patients in a year [14]. A macro-costing method was used to investigate the costs of HF using the 2014 Health Insurance Review and Assessment Service-National Patients Sample (HIRA-NPS) claims data (HIRA-NPS-2014-0067). These secondary data include the claims records for the insurance-covered costs for the inpatient, outpatient, and emergency department services, and the prescription drugs of the 3 % random sample (about 1,400,000 persons) of the entire South Korean population, which consist of the NHI and Medical Aid (MA) program enrollees. This set of data was generated by using a stratified sampling with a stratification into two sets of subgroups, namely, sex (2 strata) and age (16 strata), having a total of 32 strata [15]. In South Korea, there are two tiers for the universal health security system. The NHI program is a wage-based, contributory insurance program covering about 96 % of the population, while the MA program is a government-subsidized public assistance program for poor and medically indigent individuals [16].

### Study subjects

Adult patients with HF were defined as those aged ≥19 years who had at least one NHI or MA claim record of outpatient or inpatient services with a primary or secondary diagnosis of HF from the HIRA-NPS claims database in 2014. Due to the fact that HF in patients below 19 years old is largely attributed to congenital defects, different pathological etiologies, and lower prevalence rate than in adults, HF in patients below the age of 19 was excluded from this study [17]. Based on the literature review [7, 18–23], the diagnosis codes for HF were identified as I11.0 (hypertensive heart disease with [congestive] heart failure), I13.0 (hypertensive heart and renal disease with [congestive] heart failure), I13.2 (hypertensive heart and renal disease with both [congestive] heart failure and renal failure), I50.x (heart failure) as listed in the International Statistical Classification of Disease and Related Health Problems 10th Revision (ICD-10 codes) for a base-case group. In an effort to reflect the patient characteristics and clinical practice in South Korea, a panel consisting of three clinicians specializing in cardiology and working in tertiary-care hospitals in South Korea was consulted. They were asked about whether the diagnosis codes of HF identified from the literature are valid for Korean patients with HF. The clinician panel suggested additional diagnosis codes (I25.5 [ischemic cardiomyopathy], I42.0 [dilated cardiomyopathy], and I42.5 [other restrictive cardiomyopathy]) to comprehensively capture patients with HF from the claims records. Thus, to minimize the over- or under-specification of patients with HF, the patient group was defined in three different ways: base-case group, narrow-definition group, and extended-definition group. The patients in the narrow-definition group have the same diagnosis codes of HF as patients in the base case group, with the former having HF as the primary diagnosis. The patients in the extended-definition group included base-case patients and those who had at least one claim record of outpatient or inpatient services with a primary or secondary diagnosis of I25.5, I42.0, and I42.5 ICD-10 codes. For each patient group, healthcare utilization associated with HF was defined as claims records for outpatient, inpatient, and emergency department services, and prescriptions with the same diagnosis codes used to define the patient group.

### Estimating the economic burden of HF

The economic burden of HF was estimated both from the perspective of a payer and of the society. The costs of HF from a payer’s perspective consisted only of the NHI- or MA-covered medical costs (hereafter referred to as “NHI-covered medical costs”). Societal costs included direct medical and non-medical costs, and indirect costs defined as cost of productivity loss due to morbidity and premature death. Medical costs were divided into the NHI-covered and non-NHI-covered costs. The NHI-covered costs were estimated from the HIRA-NPS claims data. Using data from the Medical Expenditure Survey provided by NHI Service, the non-NHI-covered medical costs were estimated using the ratio of the medical costs for NHI-covered services to non-NHI-covered services for patients with heart disease. [11]. Among the non-medical direct costs, the annual per-capita transportation costs were calculated as the product of the average annual number of outpatient visits and hospital admissions due to HF per patient and the average round-trip transportation costs to visit healthcare institutions, which were obtained from the 2006 Korean National Health and Nutrition Examination Survey (KNHANES). With the assumption that at least one family member takes care of the patient during the latter’s hospitalization or a helper is hired to do such, the caregiver’s cost was calculated as the product of the average annual inpatient days per patient due to HF and the average market price for the daily charge of a helper.

The indirect costs consisted of the productivity loss costs due to morbidity and mortality and were assigned for ages under 65 years old, when people are assumed to be in the labor market. The productivity loss cost due to morbidity means the opportunity costs of time lost because of hospitalization or outpatient visits, and was calculated as shown in Eq. (1) based on the human capital approach [14, 24].

1 $$ \mathrm{Productivity}\ \mathrm{loss}\ \mathrm{costs}\ \mathrm{due}\ \mathrm{t}\mathrm{o}\ \mathrm{morbidity} = {\displaystyle \sum_i}{\displaystyle \sum_j}\left\{\left({I}_{ij} \times {D}_{ij} \times {P}_{ij}\right)+\left({O}_{ij}\times V \times {H}_{ij}\times {P}_{ij}\right)\right\} $$

*Here,*

i = age

j = gender

I_ij_ = average annual inpatient days with a diagnosis of heart failure (HF) per patient with HF by age and gender

D_ij_ = average daily income by age and gender

P_ij_ = employment rate by age and gender

O_ij_ = average annual number of outpatient visits with a diagnosis of HF per patient with HF by age and gender

V = average hours per outpatient visit

H_ij_ = average hourly wage by age and gender

The productivity loss cost due to mortality was measured based on the expected future income foregone as a result of premature death caused by HF, and was calculated as shown in Eq. (2). The age- and gender-specific number of deaths attributable to HF (ICD-10 codes of I11.0 and I50.x as a primary diagnosis) was obtained from the Annual Statistical Report of the Cause of Death by the Korean National Statistical Office (KNSO). The average annual income was derived from the age- and gender-specific average monthly income from KNSO [14].

2 $$ \mathrm{Productivity}\ \mathrm{loss}\ \mathrm{costs}\ \mathrm{due}\ \mathrm{t}\mathrm{o}\ \mathrm{mortality} = {\displaystyle {\sum}_i{\displaystyle {\sum}_j{\displaystyle {\sum}_{k=1}^n\left({N}_{ij}\times \frac{Y_{ij\left(t+k\right)}\times {P}_{ij\left(t+k\right)}}{{\left(1+r\right)}^k}\right)}}} $$

*Here,*

i = age

j = gender

k = 1, 2, …, n (difference between the life expectancy and age at the time of death)

t = age at the time of death

r = annual discount rate

N_ij_ = number of deaths caused by HF by age and gender

Y_ij(t+k)_ = average annual income at the time of (t + k) by age and gender

P_ij(t+k)_ = employment rate at the time of (t + k) by age and gender

The estimated economic burden is presented as the annual cost per patient with HF and the total national costs of patients with HF. As the productivity loss cost due to premature death was estimated based on the total number of HF deaths in the country instead of applying the individual risk of death for each patient, it was incorporated only in estimating the total national costs. All costs were expressed in 2016 monetary value.

### Sensitivity analysis

Sensitivity analyses were performed for the different approaches to define the patients with HF. The economic burden of HF estimated from the base-case patient group was compared with that estimated from the narrow- and extended-definition patient groups. Sensitivity analysis was also conducted for the mortality rate of HF using different data sources. The case definition of HF death used by the Annual Statistical Report of the Cause of Death by KNSO was based on the ICD-10 codes of I11.0 (hypertensive heart disease with [congestive] heart failure) and I50.x (heart failure) as a primary diagnosis, which under-identify HF cases compared to the case definition of the base-case patient group (ICD-10 codes of I11.0, I13.0, I13.2, and I50.x as a primary or secondary diagnosis) in this study. For the sensitivity analysis, the results from Roger’s cohort study of 4,537 HF patients older than 60 years were used [25, 26]. The one-year mortality rate (male, 20 %; female, 15 %) and the five-year mortality rate (male, 50 %; female, 46 %) were extracted from the study for the sensitivity analysis. The mortality estimates in Roger’s study were calculated through a review of the medical charts by a panel of physicians. Compared to the claims data, the mortality estimates in Roger’s study are more relevant for reflecting the precise and real clinical mortality rate of HF. For the mortality rate of the HF patients under 60 years, the same values from the Annual Report on the Cause of Death Statistics in South Korea were used in the base-case analysis.

### Subgroup analysis

To determine the impact of the disease severity of HF on the patient’s economic burden, the estimated costs across the patient subgroups were compared with the different disease severity levels. The medical costs of the HF patients younger than 65 and those older than or equal to 65 were compared. In earlier studies, it was observed that the per-capita utilization and cost of medical services to treat the same condition was significantly higher among those enrolled in the MA program in South Korea than among those enrolled in the NHI program [27, 28]. Therefore, the type of national health security program was considered a risk factor for HF patients in terms of the disease severity level and the extent of healthcare utilization. Finally, hospital admission would be a signal for a severe condition. Thus, the HF patients were divided into two groups according to experience of hospitalization, and the medical costs of the two groups were compared.

## Results

### Prevalence and healthcare utilization characteristics of the HF patients

**Fig. 1:**
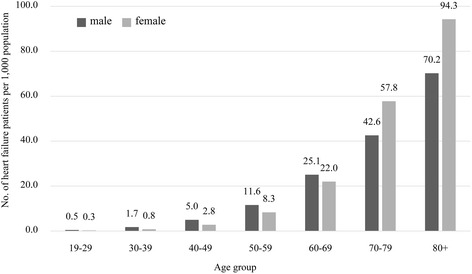
Gender- and age-specific prevalence of heart failure per 1,000 population

Table 1 presents the epidemiologic and healthcare utilization characteristics of patients with HF in Korea. According to the base-case analysis, a total of 475,019 adults (≥19 years old) in South Korea in 2014 were identified to have HF. The estimated prevalence rate of HF was 12.4 persons per 1,000 adults. The prevalence of HF was 9.2 times higher (47.8 vs. 5.2 per 1,000 population) in the elderly population (≥65 years old) than in the non-elderly population (19–64 years old). About two-thirds of the adult HF patients (65.1 %) in the country were 65 years or older. The highest proportion of HF patients was observed among those in the 70s (32.7 %), followed by those in the 60s (21.7 %), 80s or above (20.8 %), and 50s (16.0 %). Patients under 50 years old account for only 9.1 % of the adult HF patients. Overall, more women than men had HF throughout the age groups (57.7 % vs. 42.3 %). The prevalence of HF across genders differs, however, depending on the age. Up to the 60s, men have a higher prevalence of HF than women, while women have a higher prevalence of HF than men starting from the 70s (Fig. 1).

**Table 1:** Characteristics of patients with heart failure in South Korea in 2014

Characteristics	No. of patients (%) or mean (±SD)
Epidemiologic characteristics	
Estimated no. of adult patients (≥19 years old) with HF in the country	475019
Prevalence per 1000 adult population	12.4
Prevalence per 1000 population (19–64 years old)	5.2
Prevalence per 1000 population (≥65 years old)	47.8
Gender	
Male	200980 (42.3)
Female	274039 (57.7)
Age (years)	
19–64	165683 (34.9)
≥ 65	309336 (65.1)
19–29	2300 (0.5)
30–39	9332 (2.0)
40–49	31230 (6.6)
50–59	75526 (16.0)
60–69	102823 (21.7)
70–79	15584 (32.7)
≥ 80	98623 (20.8)
Types of health security program enrolled in	
NHI	426257 (89.7)
MA	46962 (9.9)
Healthcare utilization characteristics^a^	
Hospitalization rate (%) of adult patients with HF	12.1
Hospitalization rate (%) of patients (19–64 years old) with HF	7.1
Hospitalization rate (%) of patients (≥65 years old) with HF	14.8
No. of outpatient visits per patient	5.01 (±7.33)
No. of hospital admissions per patient	0.24 (±1.07)
No. of inpatient days per patient	3.83 (±26.08)
No. of inpatient days per hospital admission	15.76 (±11.79)
No. of hospital admissions per patient with at least 1 admission	2.01 (±2.42)
No. of inpatient days per patient with at least 1 admission	31.70 (±68.96)

All characteristics listed correspond to results for adult patients aged 19 years or above unless otherwise specified

*HF* heart failure, *NHI* National Health Insurance, *MA* Medical Aid, *SD* standard deviation

^a^All healthcare utilizations were defined as outpatient visits or hospital admissions with a primary or secondary diagnosis code of heart failure (ICD-10 codes of I11.0, I13.0, I13.2, and I50.x)

On average, the patients with HF had 5.01 outpatient visits, 0.24 hospital admissions, and 3.83 inpatient days for HF treatment in a year. About 12 out of 100 HF patients had hospital admissions due to HF, with 15.76 inpatient days per admission. As age increased, the probability of hospitalization increased: 14.5 % of the elderly patients aged 65 or older had hospitalization due to HF while only 7.1 % of the non-elderly patients had hospitalization due to HF. For the patients with at least one episode of HF-associated hospitalization, the average number of annual hospitalizations due to HF, including the initial hospitalization, was 2.01, and the total number of inpatient days was 31.70 days.

### Socioeconomic burden of HF patients

The average annual medical expenditure for NHI-covered services spent by individual patients to treat HF was USD 868.2 in 2014 (Table 2). From the societal perspective, the average spending of each patient in 2014 was USD 1,414.0. The total national NHI-covered medical expenditure for the treatment of HF across South Korea amounted to USD 412.4 million. While only 12 % of the patients with HF were hospitalized (Table 1), the medical expenses for inpatient services accounted for 53.4 % of the NHI-covered medical expenditure. From the societal perspective, the economic burden of HF in the country was estimated to be USD 752.8 million. Medical costs accounted for the biggest portion of the national burden (68.5 % = 54.8 + 13.7 %), followed by the caregiver’s cost (13.2 %), the productivity loss costs due to premature death (10.8 %) and morbidity (4.2 %), and transportation costs (3.4 %).

**Table 2:** Economic burden of heart failure in South Korea in 2014

	Per-capita cost, USD		Total national cost, million USD	
	Perspectives		Perspectives	
	Payer	Society	Payer	Society
Total costs (direct and indirect costs)	868.2 (100.0 %)	1414.0 (100.0 %)	412.4 (100.0 %)	752.8 (100.0 %)
Direct costs		1347.8 (95.3 %)		640.2 (85.0 %)
NHI-covered medical costs	868.2 (100.0 %)	868.2 (61.4 %)	412.4 (100.0 %)	412.4 (54.8 %)
Inpatient services (including prescription drug costs)	463.3 (53.4 %)	463.3 (32.8 %)	220.1 (53.4 %)	220.1 (29.2 %)
Outpatient services (including prescription drug costs)	404.9 (46.6 %)	404.9 (28.6 %)	192.3 (46.6 %)	192.3 (25.6 %)
Prescription drug costs^a^	285.7 (32.9 %)	285.7 (20.2 %)	135.7 (32.9 %)	135.7 (18.0 %)
Non-NHI-covered medical costs	-	217.0 (15.3 %)	-	103.1 (13.7 %)
Non-medical costs	-	262.5 (18.6 %)	-	124.7 (16.6 %)
Transportation costs		53.9 (3.8 %)		25.6 (3.4 %)
Caregiver’s costs		208.7 (14.8 %)		99.1 (13.2 %)
Indirect costs	-	66.3 (4.7 %)	-	112.6 (15.0 %)
PL due to morbidity	-	66.3 (4.7 %)	-	31.5 (4.2 %)
PL due to premature death	-	-	-	81.1 (10.8 %)

All costs were expressed in 2016 monetary value (1 USD = 1,150 Korean won)

*USD* U.S. dollar, *NHI* National Health Insurance, *PL* productivity loss

^a^Prescription drug costs were calculated as the sum of inpatient and outpatient prescription drug costs

Sensitivity analyses were performed with varying approaches to define patients with HF (Table 3). When HF patients were defined in the most conservative way (using the narrow definition of patients), the total number of patients was reduced by 46.1 %, from 475,019 to 256,241 patients. Also, the total national cost was reduced by 58.7 %, from USD 752.8 to 311.1 million. On the other hand, the impact of using the extended definition of HF patients was marginal, increasing the total number of patients by only 1.1 %, from 475,019 to 498,783 patients, and increasing the total national cost by only 9.1 %, from USD 752.8 to 822.0 million. The impact of using different data sources for the mortality rate of HF was also examined through sensitivity analysis (Table 3). As both the one- and five-year mortality rates were higher than the mortality rate reported by the Annual Statistical Report on the Cause of Death, the total national costs were increased to USD 878.4 and 1,085.6 million, respectively.

**Table 3:** Sensitivity analysis results by varying the definition of patient group and mortality rate

	Per-capita cost, USD	Total national cost, million USD
Base case (*N* = 475019 patients)	1414.0	752.8
Definition of patient		
Narrow definition (*N* = 256241 patients)	948.8	311.1
Extended definition (*N* = 498783 patients)	1560.5	822.0
Mortality rate		
1-year mortality	-	878.4
5-year mortality	-	1085.6

*USD* U.S. dollar

All costs were expressed in 2016 monetary value (1 USD = 1,150 Korean won) and estimated from a societal perspective

The estimated medical costs substantially varied across the subgroups that were examined in this study (Table 4). The elderly patients with HF aged 65 or above spent about 1.6 times more for NHI-covered medical services than the non-elderly patients aged 19–64 years old. The annual per-capita NHI-covered medical cost for treating HF was 1.6 times higher for the patients enrolled in the MA program than for those enrolled in the NHI program. Finally, compared to the HF patients who had not experienced hospitalization associated with HF, those with at least one episode of hospitalization due to HF showed 9.7-fold higher NHI-covered medical costs for treating HF.

**Table 4:** Economic burden of heart failure by subgroup

	Annual per-capita NHI-covered medical costs, USD	Ratio of costs
By age		
19–64 years old	629.5	1.0
≥ 65 years old	996.0	1.6
By type of health security program enrolled in		
NHI	821.1	1.0
MA	1310.9	1.6
By experience of hospitalization		
Not hospitalized in 2014	422.9	1.0
Hospitalized in 2014	4104.4	9.7

*USD* U.S. dollar, *NHI* National Health Insurance, *MA* medical aid

All costs were expressed in 2016 monetary value (1 USD = 1,150 Korean won) and estimated from the payer’s perspective

## Discussion

In this study, the prevalence and economic burden of the adult HF patients in South Korea were estimated using the nationally representative HIRA-NPS data, which cover the insurance claims records of the 3 % random sample of the entire population in the country. Based on the most recent HIRA-NPS data (2014), the analysis in this study revealed that the estimated prevalence rate of HF was 1.24 %. This figure is similar to the prevalence rate in other countries, such as USA, UK, Italy, and Denmark, which was reported at approximately 1–2 % [2, 9]. As observed in other countries [29, 30], it was confirmed that the prevalence of HF also increases with age in South Korea. The elderly aged 65 or older showed a 9.2-fold higher prevalence of HF than the non-elderly population aged 19–64.

Due to the fact that HF is usually accompanied by many underlying diseases, it is often difficult to identify the correct cases of HF based on the diagnosis codes from the insurance claims data. To improve the validity of the case definition, different approaches to defining HF cases were carried out in this study: the base-case, narrow-definition, and extended-definition approaches. While the estimated number of HF patients identified according to the extended definition of HF increased only by 1.1 % from the base-case patients, it decreased by 46.1 % when the narrow definition of HF patient was used. The difference in the method of identifying HF patients between the narrow and base-case definitions comes from whether only the primary diagnosis was used or both the primary and secondary diagnoses were used to define the cases using the same ICD-10 codes (I11.0, I13.0, I13.2, and I50.x). As a result of the use only of the primary diagnosis in the narrow-definition group, the total number of HF patients in the country was reduced to about half of the base-case patient groups, which used both the primary and secondary diagnoses to define HF patients. To determine which approach is more valid for capturing HF cases, we examined the primary diagnoses of those identified as HF patients based on their secondary diagnosis. About 75 % of the primary diagnoses of the patients were conditions related to HF, such as hypertension (33.6 %), angina pectoris (10.3 %), non-insulin-dependent diabetes mellitus (9.4 %), atrial fibrillation and flutter (6.6 %), chronic kidney disease (6.2 %), and chronic ischemic heart disease (2.7 %) (Table 5). As a result, it appears that the narrow definition would cause under-specification for HF cases. Thus, based on the base case and the extended definition of cases, it is reported that the annual economic burden of HF from the societal perspective ranges from USD 1,414.0 to 1,560.5 for individual patients, and from USD 752.8 to 1,085.6 million for the entire country.

**Table 5:** Distribution of primary diagnoses of base-case patients identified as having heart failure based on the secondary diagnosis

Rank	ICD-10 code	Description	Percent
1	I10	Essential (primary) hypertension	33.59
2	I20	Angina pectoris	10.26
3	E11	Non-insulin-dependent diabetes mellitus	9.41
4	I48	Atrial fibrillation and flutter	6.61
5	N18	Chronic kidney disease	6.21
6	I25	Chronic ischemic heart disease	2.70
7	I42	Cardiomyopathy	1.97
8	I21	Acute myocardial infarction	1.81
9	I63	Cerebral infarction	1.58
10	E14	Unspecified diabetes mellitus	1.27
Total			75.41

As mentioned earlier (in the Background section), patients with HF have a high risk of hospitalization. This is reflected in the cost estimation results of this study. The inpatient services accounted for 53.4 % of the NHI-covered medical costs for the individual patients with HF (Table 2). Compared to other chronic diseases that are common among the elderly such as hypertension (18.3 %), diabetes (36.4 %), rheumatic arthritis (18.9 %), and respiratory disorder (49.5 %) including asthma, COPD, and emphysema, HF (53.4 %) incurred higher spending for inpatient services in 2014 [24]. Also, patients who had experienced hospitalization with HF incurred about ten times higher NHI-covered medical costs (Table 4). These findings suggest that effective intervention to prevent hospital admission would be a critical component of the disease management strategy for patients with HF to minimize the economic and clinical burden of HF.

The higher costs occurred among the MA patients than the NHI patients. This was explained by the differences in hospitalization rate and proportion of the elderly between the two groups. The proportion of the HF patients experiencing hospitalization is approximately 1.7 times higher in the MA patients (19.2 %) than the NHI patients (11.3 %). In addition, the higher proportion of the elderly aged 70 years and above is observed among the MA patients (64.2 %) than the NHI patients (52.4 %).

As in most other illnesses, the elderly patients seemed to require more healthcare resources to treat HF compared to the younger patients. Those aged 65 or above in this study spent about 1.6 times more NHI-covered medical costs than those aged 19–64. The higher medical spending among the elderly group may be partly attributable to the higher risk of hospitalization that comes with aging. The hospitalization rate of those aged 65 or above among the study subjects was about twice that of those aged 19–64 (14.8 % vs. 7.1 %).

With the NHI claims data recently made available to the general public in South Korea for research purposes, there are ongoing researches on the cost of the illness, but most of the researches on HF are focused on primary diagnoses or with a narrowly defined scope of HF, posing restrictions on the accurate estimation of the cost of HF. In contrast, some studies have been conducted in hospital settings. According to a previous study conducted using electronic medical chart review for HF patients from tertiary-care hospitals, the average cost per hospitalization with HF was estimated to be approximately USD 7,000 [31], which is 1.7 times higher than what was estimated in this study (USD 4,140). The difference in the estimated hospitalization cost is attributable to the fact that the previous study limited the study subjects to the patients with acute HF in the tertiary hospitals.

This study has the potential risk of underestimating the cost of HF for the following reasons. First, in calculating the transportation costs incurred for an outpatient visit or for hospital admission, only the patient’s transportation costs were included. It was possible, however, that at least one caregiver accompanied a patient when he/she visited the hospital on an outpatient basis or was admitted to a hospital because about 64 % of HF patients are aged 65 years or above who require assistance from others. Second, based on the human capital approach, the productivity loss costs due to morbidity and premature death were accounted for only for those under 65 years old, with the assumption that those above 65 years old are no longer productive and no longer contribute to the society. This leads to underestimation of costs as well as the ethical problem of not ascribing any value to the latter years of life. Third, to overcome the tendency to underestimate premature death costs caused by under-specified HF death from the national death statistics, sensitivity analysis was conducted using the one- and five-year mortality rates of HF patients based on existing relevant literature. However, the use of the literature value was limited to only those who were ≥60 years old. Thus, there is still a possibility of underestimating premature death costs for cases of patients under 60 years old.

This study also has a potential to overestimate the economic burden of HF. In calculating the productivity loss costs due to morbidity and mortality, a 100 % employment rate was assumed for those under 65 years old. This does not imply that all the HF patients are employed. The rationale for this assumption is that the time of the HF patients who are not part of the labor market should not be undervalued compared to that of patients who are part of the labor market. If it is insisted that productivity loss can occur only for those who are part of the labor market, the proposed approach may overestimate the costs.

## Conclusions

This study presented the extent of the economic burden attributable to heart failure (HF) in the South Korean society. The prevalence rate of HF in 2014 was 12.4 out of 1000 adults, while the annual socioeconomic cost of HF was estimated to be 752.8 million USD. The onset of HF is positively correlated with aging. Due to the extended life expectancy, the prevalence of HF, accumulated from the long-term survivors, is expected to grow continuously. As such, HF has drawn special attention in the existing aging society. In this study, HF patients who were older and had a history of prior hospitalization with HF as well as an indigent status (i.e., enrolled in the Medical Aid [MA] program) were shown to be at high risk to spend more for healthcare to treat their HF. An effective disease management protocol should be employed to target such patient group.

## Abbreviations

HF: Heart failure
HIRA-NPS: Health insurance Review and Assessment Service-National Patients Sample
ICD: International Statistical Classification of Disease
KNHANES: Korean National Health and Nutrition Examination Survey
KNSO: Korean National Statistical Office
MA: Medical aid
NHI: National Health Insurance
